# Non-returning factors from an interview survey of 16 residents of Katsurao village 12 years after the Fukushima nuclear power plant accident

**DOI:** 10.1093/rpd/ncae107

**Published:** 2024-11-14

**Authors:** Naomi Ito, Isamu Amir, Nobuaki Moriyama, Ayako Furuyama, Mika Sato, Chika Yamamoto, Tianchen Zhao, Masaharu Tsubokura

**Affiliations:** Department of Radiation Health Management, School of Medicine, Fukushima Medical University, 1 Hikariga-oka, Fukushima 960-1295, Japan; Department of Radiation Health Management, School of Medicine, Fukushima Medical University, 1 Hikariga-oka, Fukushima 960-1295, Japan; Department of Public Health, School of Medicine, Fukushima Medical University, 1 Hikariga-oka, Fukushima 960-1295, Japan; Health Promotion Center, Fukushima Medical University, 1 Hikariga-oka, Fukushima 960-1295, Japan; Department of Health Nursing of International Radiation Exposure, Graduate School of Medicine, Fukushima Medical University, 1 Hikariga-oka, Fukushima 960-1295, Japan; Department of Radiation Health Management, School of Medicine, Fukushima Medical University, 1 Hikariga-oka, Fukushima 960-1295, Japan; Department of Radiation Health Management, School of Medicine, Fukushima Medical University, 1 Hikariga-oka, Fukushima 960-1295, Japan; Department of Radiation Health Management, School of Medicine, Fukushima Medical University, 1 Hikariga-oka, Fukushima 960-1295, Japan

## Abstract

People generally wish to return home after being evacuated due to disaster situations. Evacuation orders have now been lifted in the Fukushima region following the nuclear accident in 2011, and the Japanese government is promoting a return policy. However, many residents who wish to return home remain unable to and continue living in evacuation sites or other areas. Sixteen residents of Katsurao village were interviewed after evacuation orders were lifted in 2016 who have not yet returned. Concerns were cited regarding radiation, prolonged evacuation, health problems, buying a house in the evacuation area and schooling. The problems identified were primarily due to the rapid ageing and decline of the regional population, reflecting similar issues throughout Japan. In particular, health problems and intention to return were thought to be closely related. Over 10 y have passed since the evacuation, and many residents have experienced familial separation and divided living situations.

## Introduction

People generally want to continue living in a familiar region whenever possible [1]. This idea of ageing in a specific environment represents a central concept of community living [2]. In particular, it can relate to identity through human relationships, roles and independent living in older adults [3]. However, it may not be possible to age in an environment where factors such as disasters or illnesses complicate matters. Taking measures to facilitate ageing in an environment following such events is a significant public health concern.

**Figure 1:**
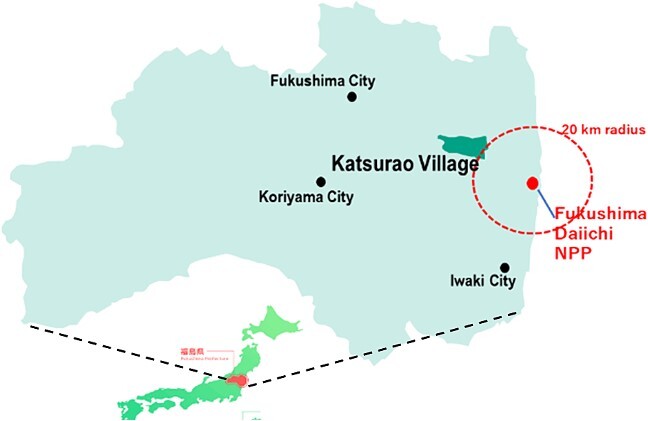
The location of Katsurao village.

After the Fukushima nuclear power accident in March 2011, all residents of Katsurao village, located within a 20–30 km radius of the plant, were evacuated. Evacuation orders were lifted for most of the village in June 2016, and the Japanese government has since been promoting a return policy (Fig. 1).

Katsurao village had a population of about 1500 at the time of the nuclear accident. In 2016, it became certain that the annual cumulative radiation dose would be below 20 mSv, and evacuation orders were lifted. Infrastructure necessary for daily life was also restored, and in particular, schools in the village reopened in 2018. However, only about 30% of the residents have returned 7 y after the evacuation order was lifted. According to a survey by the Reconstruction Agency, 14% of the residents said: ‘they want to return in the future’, while nearly 50% said they were ‘undecided’ or ‘would not return’ [4]. The research question here is the common factor behind the reasons for not returning, which could contribute to the future support of the residents.

This report summarises interviews with former residents of Katsurao village who were evacuated following the 2011 Fukushima nuclear accident and have not yet returned. The study aimed to understand the living conditions that the residents experienced after the disaster and identify the factors that prevented them from returning home. This would provide insight into residents’ challenges and the most effective support measures to help them return.

## Methods

The subjects were registered residents of Katsurao village, with whom the first author had the opportunity to speak while assisting with healthcare-related initiatives. Interviews were conducted with 16 residents who evacuated following the evacuation order in March 2011. They have not yet returned to Katsurao Village and live outside it. This number corresponds to 2% of the 801 non-returning village residents [5].

The survey period was from October 2022 to June 2023. Semi-structured individual interviews were conducted in and outside the village. The interview time was approximately 30 minutes per person.

The following were the two main questions: [1] Why have not you returned to Katsurao? [2] What is your current living situation?

The responses were analysed, and elements that could be used to understand the current living situations of the participants were identified and categorised. Elements considered to be important were extracted from the narratives, categorised and named based on similarities in nature. In addition, the relationships between the categories generated were represented in a results chart and storyline.

### Ethical considerations

The study’s significance, purpose and method were explained to each participant before their interview, and written informed consent was obtained. Privacy protection was considered by ensuring anonymity. The study was conducted in accordance with the Declaration of Helsinki, and was approved by the Fukushima Medical University Ethics Committee (Approval #2019-269; date: 20 September 2022).

## Results

### Participants

There were 16 participants in total (eight men and eight women). One participant was in their 30s, two were in their 40s, two were in their 50s, two were in their 60s, eight were in their 70s and one was in their 90s.

### Results

The first author classified the data, and the two co-authors reviewed it.

**Figure 2:**
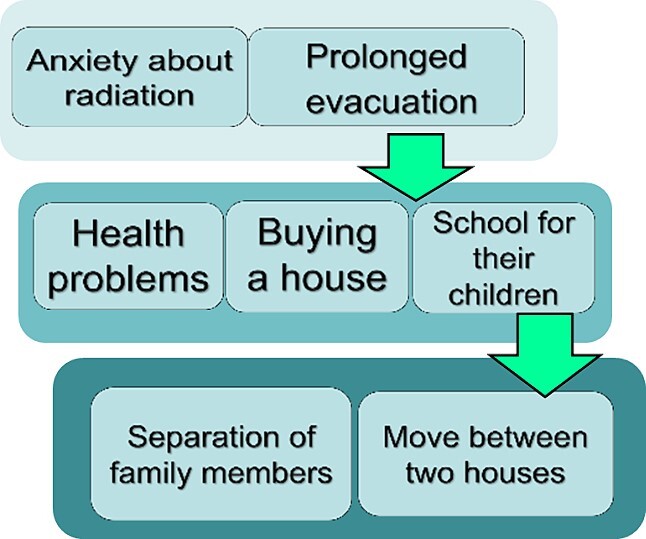
Structural chart of the seven major response categories.

Seven categories were identified: ‘prolonged evacuation period’, ‘concerns about radiation’, ‘health issues’, ‘purchasing a house’, ‘schooling for children’, ‘separated families’ and ‘houses in two locations’. On the basis of the research questions, the causal relationships and timelines among the categories were considered and charted (Fig. 2).

All 16 participants reported that they were facing a ‘prolonged evacuation period’ and did not know when they would be able to return:

‘I didn’t know when I was going to go back, and my whole family moved to Iwaki’ (Female in her 70s).

There were 11 participants who ‘purchased a house’. Owing to the prolonged evacuation period, they purchased houses within the new areas they had been evacuated to begin rebuilding their lives:

‘Considering my husband’s hospital visits, I thought it would be better to buy a house in a convenient place, so I bought it’ (Female in her 70s).

‘The lifting of the evacuation order was delayed in my part of the village, and no one has returned, so I bought a house because I wanted to live in Koriyama’ (Female in her 70s).

Ten participants reported going back and forth between ‘houses in two locations’. Of these, there were several types

(i) Previously, a large family with three generations lived together. The older generation returned to the village, and the younger generation bought a house in the evacuation area.

(ii) The whole family owned a house in the evacuation destination and their original house in the village. They regularly commute to the village to work on the farm.

(iii) While purchasing and maintaining a house in the area they had been evacuated, they lived in a facility or apartment complex in search of the services they sought:

‘I grow vegetables in the field, so I go to the village every day from Funabiki’ (Female in her 70s).

‘I stay at home in the village during the day and go home at night in Miharu’ (Female in her 60s).

Six interviewees reported being concerned about ‘schooling for children’. They prioritised maintaining their children’s educational stability by finding schools in the evacuation areas.

‘My grandson has made lots of friends at the school he was evacuated, and we can’t all go back to the village’ (Male in his 70s).

‘It’s too far from the village to go to school’ (Female in her 40s).

Five participants reported ‘health problems’. Some came to require continuous support such as hospital visits, treatment, and nursing care while living outside the village, and some preferred a more convenient lifestyle due to old age.

‘My husband entered an institution at the evacuation site, and I visit him from time to time’ (Female in her 70s).

‘During the evacuation, my dialysis treatment started, and I do not drive anymore, so it is difficult for me to go to the hospital three times a week from the village’ (Male in his 70s).

Five of the participants responded in the affirmative to ‘families separated’. Some participants had previously lived in multigenerational homes with three generations under one roof. However, following the evacuation, the oldest generations returned while the younger ones adapted to employment or schooling in their new locations:

‘My son went back to his house in the village for work, my grandchildren are in school at the evacuation site, and my wife and I built a house at the evacuation site. We all became separated’ (Male in his 70s).

Three people reported ‘concerns about radiation’, including those who had been against nuclear power before the accident, as well as those who had concerns surrounding their children being exposed to radiation:

‘I’ve been worried about nuclear power for a long time, and I thought I’d never go back’(Female in her 90s).

## Discussion

This survey revealed a series of typical secondary effects of the disastrous Fukushima accident that residents currently face in the areas where evacuation orders have since been lifted. The impact on residents following any nuclear power accident is diverse and includes considerations not only related to radiation exposure but also lifestyle and social environment changes [6]. This report captures one aspect of the impact on residents’ lives over medium- to long-term periods, as various environmental changes occur during rebuilding.

Prolonged evacuations can affect many aspects of the situation. Five years have passed since the large-scale evacuation order was lifted for Katsurao village. In many cases, people with health problems or young families with children decided not to return. In particular, health issues were among the significant factors when evacuees considered their intentions to return. Health issues that arose during the prolonged evacuation period were often addressed by social resources that the village lacked, such as medical and welfare services in the regions where the residents were relocated. It has, therefore, become difficult for them to return to their original village and continue to maintain their quality of life [7]. It was also learned that, as time passed, many residents could rebuild their lives in the new regions they had been evacuated. They bought houses and chose new schools for their children in these regions. Fear of radiation exposure may also have driven children and their parents away from Katsurao village. Many of the residents’ children had spent most of their lives in the new areas where their families had been evacuated. As a result, even if the effects of radiation were ruled out and they could return home, some did not see any point in returning to Katsurao village. It may be effective to devise a reopening date for schools and announcements to encourage the return of young people with children.

As a result, family structures have changed, and family separation has occurred in some families—particularly those where three generations formerly lived together before the disaster. In some cases, these previous family relationships have become dysfunctional. An increasing number of people now rely on public nursing care services to live outside their villages instead of the support they previously received from their families [8]. Many residents adopted new ways of life following the Fukushima accident and began living in two locations, commuting between their former and new homes [9]. These findings suggest that a phenomenon particular to post-radiation disasters may have occurred in this area, as houses and land have remained intact but unoccupied. This contrasts with situations following other disaster types, such as earthquakes and tsunamis, which often cause physical losses of homes.

There are several limitations to this study. Half of the interviewees are in their 70s. Because residents are scattered and opportunities to meet residents are limited, interviews have been conducted through group health checkups and home visits. Additionally, as the number of participants was small and the survey was limited to the Katsurao area, we plan to conduct interviews in other regions in the future to understand the affected area after the nuclear disaster.

## Conclusions

As residents of Katsurao village began rebuilding their lives in new locations before the evacuation order surrounding the Fukushima region was lifted, the number of factors preventing them from returning home began to increase. The prolonged evacuation period had a significant impact. Areas where evacuation orders have been lifted following Fukushima’s nuclear accident are facing rapid population decline and ageing. Apart from encouraging the general return of the resident population, it is crucial to strengthen health-related support systems within such villages. Many residents are facing new challenges regarding living in two locations and having their families separated as more and more time passes following the evacuation. These factors continue to present new challenges to evacuees and their families.
